# Supplementary material for: Utilization of cervical cancer screening and associated factors among women in Debremarkos town, Amhara region, Northwest Ethiopia: Community based cross-sectional study
Source: PLoS One. 2020 Apr 7;15(4):e0231307. doi: 10.1371/journal.pone.0231307 (PMC7138328; doi:10.1371/journal.pone.0231307)
Supplement: Attachment — Submitted filename: Response to Reviewers.docx [file pone.0231307.s001.docx]

**Response to Reviewers**

**Dear reviewer**

Thank you for your constructive comments on our manuscript. Again your suggestion towards title modification is also right.

- But from the outset, our outcome variable is "utilization of cervical cancer screening ". Knowledge and attitude are our independent variables so that variables that influence knowledge and attitude not fully included in the questionnaire. Again, during analysis, we have not used Likert scale analysis for attitude since we have used it as an independent variable. Because of these reasons, we have decided to put the original title (Utilization of Cervical Cancer Screening and Associated Factors among Women in Debremarkos town, Amhara Region, Northwest Ethiopia: Community based cross-sectional study).
- We have used a 3.5 % margin of error to increase sample size and the rule of thumb says if ‘P-value' less than 20% and greater than 80% we can use margin of error from 2% to 4%.
- Since it has yes/no outcome, the utilization of cervical cancer screening is analyzed by using binary logistic regression.
- The knowledge question was changed to binary (1=yes and no=0). Then calculated the mean value and women who answered knowledge questions score of mean value or above were considered as knowledgeable.
- The attitude question was also changed to binary (1=agree and 0=disagree + indifferent). Then calculated the mean value and women who answered attitude questions a score of mean value or above were considered to have a favorable attitude.
- Self employee means individuals who are doing their own small business and private employee means individuals who are salaried in the private sector. As you have recommended, the definitions are described in the bracket.
- History of MSPs, History of smoking, and SDs are adapted from different literatures. History of MSPs and History of smoking have been operated. They are relevant because different they are associated with cervical cancer screening in different studies.
- In attitude, the first two questions seem similar in concept but, women may not think as they are susceptible to cervical cancer even though they think as others are susceptible. That is why the two questions have different figure (25.2% versus 24.6% )
- We have used “Cervical cancer screening can be practiced” to say ‘**All eligible mothers can have cervical cancer screening without complication’**
- “**Cervical cancer is a killer disease**". We know as it is a killer disease but since it is a community-based study, women may not know whether it is a killer disease or not. So, it may be appropriate.
- **A family disease** means a disease that can be transmitted genetically.
- **Hazardous to your health** means cervical cancer may be dangerous which complicates women’s life.
- **Wellbeing** is used to show the benefit of cervical cancer screening to health. Even though it is a general question to assess the attitude of women towards cervical cancer screening. Women may think screening by itself induces cervical cancer.
- We have cells with a single value. The main reason is there is a small number of data that were found based on ethnicity (after merging of Oromo and Gurage, only one person was utilized screening) so, we could not merge it further.
