# Supplementary material for: Utilization of cervical cancer screening and associated factors among women in Debremarkos town, Amhara region, Northwest Ethiopia: Community based cross-sectional study
Source: PLoS One. 2020 Apr 7;15(4):e0231307. doi: 10.1371/journal.pone.0231307 (PMC7138328; doi:10.1371/journal.pone.0231307)
Supplement: Attachment — Submitted filename: utilization of cervical cancer screening1 (1).docx [file pone.0231307.s002.docx]

Utilization of Cervical Cancer Screening and Associated Factors among Women in Debremarkos town, Amhara Region, Northwest Ethiopia: Community based cross sectional study

**Bewket Yeserah Aynalem^1^, Kiber Temesgen^2^, Mihretu Molla Eniyew^2^**

1 Department of midwifery, Debremarkos University, Debremarkos, Ethiopia

2 Department of midwifery, University of Gondar, Gondar, Ethiopia

# Abstract

## Introduction

Cervical cancer is the most common disease among all malignancies for woman worldwide with 266 000 deaths every year. Even though there is a proved importance of cervical cancer screening, the death of women due to cervical cancer in Ethiopia is high. We therefore did this study to investigate utilization of cancer screening, and its associated factors among women in Debremarkos town, Amhara region, Ethiopia.

## Methods

Community based cross-sectional study was conducted on women from 30-49 years in Debremarkos town, from July 1 to August 30, 2018. Multistage sampling procedure was used to select 822 women in to the study. We used EPI info version 7 for data entry and SPSS version 24 software for cleaning and analysis. Bivariable and multivariable logistic regressions were performed to identify factors associated to the outcome variable with 95% CI and p-value less than 0.05 was set for association.

## Result

The study revealed that 44 (5.4 %) of the women have been screened for cervical cancer. Women’s age [AOR:3.126(1.246,7.845)], marital status (AOR:3.41(1.299,8.972)], educational status ([AOR:7.27(2.07,25.513)],[AOR: 4.578(95% CI: 1.19, 17.65)]), age at first sex [AOR:3.021(1.84,4.97)], history of multiple sexual partners [AOR:2.51(1.040, 6.06)], history of sexually transmitted disease [AOR:4.04(1.68, 9.72),], knowledge [AOR:4.02(2.07,7.77)] and attitude [AOR:3.23(2.52,4.12)] were significant factors for utilization of cervical cancer screening.

## Conclusion

This study showed the magnitude of utilization of cervical cancer screening is low. Women’s age, marital status, educational status, age at first sex history of multiple sexual partners and sexually transmitted disease, knowledge and attitude were important factors of screening.

# Introduction

Cervical cancer is the most common disease among all malignancies for woman worldwide (1). About 266000 deaths were occurred due to cervical cancer all over the world and 90% of cases occurred in Low and Middle-Income Countries (LMICs); the highest was occurred in Sub Saharan Africa (SSA) where cervical cancer is the leading killer of women(2). in Ethiopia,70,95 cases and 4732 death of cervical cancer was occurred every year (3).

Cervical cancer screening has been proven to be very effective prevention strategy for cervical cancer (1, 4, 5).

The United States Preventive Services Task Force (USPSTF), the American Cancer Society (ACS) World Health Organization (WHO) guidelines state screening for cervical cancer once with in life time reduces significantly the risk of mortality from cervical cancer and incidence of advanced cervical cancer (6-8).

The WHO recommended age for cervical cancer screening should be limited to women in the age of 30 to 50 years, since there is an evidence on younger age women with milder degree of lesion spontaneously recover to normal (5, 9, 10).

Ethiopia adopted the WHO guideline and advised women to start cervical cancer screening at age of 30-49 years at least one to three years interval with the approach of see and treat through Visual Inspection under Acetic acid (VAI) as screening strategy and cryo-therapy as a treatment method (1, 4, 5, 11).

Despite the importance of screening, high incidence of cervical cancer is still a big problem and a major cause of morbidity and mortality of women in LMICs especially in SSA (12, 13) and the lowest cervical cancer screening rate found in Ethiopia which accounts below 1% (14).

Therefore, the main aim of this research is to assess utilization of cervical cancer screening and associated factors among women at Debremarkos town.

# Methods

A community based cross-sectional study was conducted at Debremarkos town, Amhara region, Northwest Ethiopia from July 1 to August 30, 2018. Debremarkos town is capital city of East Gojjam Zone that comprises 7 kebeles. There is one referral hospital which gives cervical cancer screening service. The study populations were all women with the age 30-49 years who were residents of Debremarkos town during the study period in the selected kebeles.

Sample size for prevalence was determined based on single population proportion formula assumption. Expected proportion of cervical cancer utilization (19.8%) from previous study in Ethiopia at Mekelle town(15) and 3.5% confidence limit (margin of error) was used.

$$\mathrm{initialsamplesize}={(Z\frac{a}{2})}^{2}*\frac{p(1-p)}{w2}={1.96}^{2}*\frac{0.198\left( 1-0.198 \right)}{({0.035)}^{2}}=498$$

With considering design effect 1.5 since it had two stages and the sample size was calculated as 498*1.5= 747 then non-response rate was also considered to be 10%and 747*0.10=75.Then the final sample size was747+75=822.

A multistage sampling technique was used and three out of the seven kebeles was selected by lottery method. The total sample size calculated for the study 822 was distributed to the kebeles in the town using proportional allocation based on the estimated number of households.

To assure the data quality, data were collected with face to face interview by three trained BSc Midwives after one day data collection training was given to them together with their three MSc holder supervisors. The questionnaire was structured and pre-tested which was first prepared in English and translated to local language and then again translated back to English. Pretest was conducted on 42 women of the sample size in other than the study area and necessary correction on tool was employed accordingly. Epi Info version 7 software was used for data entry and SPSS version 24 for used for analysis. Bivariate logistic regression was employed to identify association between independent and dependent variables. Variables having P-value less than 0.2 in the bivariate logestic regression analysis was fitted in to the multivariable logistic regression model. The 95% confidence interval of odd ratio was computed and variable having P-value less than 0.05 in the multivariable logistic regression analysis was considered as statistically significant.

Ethical clearance was obtained from the Ethical Review Committee of School of Midwifery, under the delegation of the institutional review board of University of Gondar. Ethical clearance and formal letter was also obtained from University Gondar School of Midwifery and was submitted to Debremarkos health office and permission was obtained. Finally, written informed consent was also obtained from each study participants.

**Results**

## Socio demographic characteristics of the respondents

A total of 822 study participants were included in the analysis with the response rate of 100%. The participants’ ages ranged from 30 to 49 and the mean age of respondents was 36.81 years with ±5.14SD. With age distribution, 592 (72%) study participants belonged to from age 30 to 39 years; with marital status distribution 638 (77.6%) study participants were married. In this study, 789 (96%) study participants were Christian and 809 (98.4%) study participants were Amhara. Similarly, from the total participants, 308 (37.6%) were self-employed; 231 (28.1%) study participants had no formal education. Two hundred ninety-three (35.6%) of the respondents have their monthly income between 1601-2699 Ethiopian Birr (Table 1).

Table 1: Socio-demographic characteristics of women (n=822) age 30-49 in Debremarkos town, Northwest Ethiopia, 2018

| **Variable** | **Frequency** | **Percent** |
| --- | --- | --- |
| **Age of mothers**  30–39  40–49 | 592  230 | 72  28 |
| **Marital status**  Married  Others* | 638  184 | 77.6  22.4 |
| **Religion**  Christian  Muslim | 789  33 | 96  4 |
| **Educational status**  No formal education  Primary education  Secondary education  College and above | 231  180  212  199 | 28.1  21.9  25.8  24.2 |
| **Ethnicity**  Amhara  Others** | 809  13 | 98.4  1.6 |
| **Occupation**  House wife  Self-employee  Private employee  Government employee | 149  309  192  172 | 18.1  37.6  23.4  20.9 |
| **House hold income**  <900  900–1600  1601–2699  >=2700 | 166  201  293  162 | 20.2  24.5  35.6  19.7 |

* ^Single, divorced and widowed, **^ ^Oromo and Guragie^

## Reproductive and behavioral characteristics of the respondents

From 822 study participants, 201 (24.5%) started sexual intercourse for the first time with the age of sixteen or below. Women who had a history of multiple sexual partners (MSPs) were 461 (56.1%). And about 129 (15.7%) had a history of the sexually transmitted disease (STD). Six hundred eighty-nine (83.8%) of the study participants had used modern family planning method at least for one year. Six hundred sixty-three (80.7%) got birth at least once; from this about 558(84.2%) were para five and below. Around ninety-seven (11.8%) of respondents had a family history of cervical cancer and 17 (2.1%) of the respondents had also smoking history (Table 2).

Table 2: Reproductive characteristics of women (n=822) age 30-49 in Debremarkos town, Northwest Ethiopia, 2018

| **Variable** | **Frequency** | **Percent** |
| --- | --- | --- |
| **Age at first sex**  <=16  >16 | 201  621 | 24.5  75.5 |
| **Multiple sexual partner**  No  Yes | 461  361 | 56.1  43.9 |
| **History of smoking**  No  Yes | 805  17 | 97.9  2.1 |
| **History of STD**  No  Yes | 693  129 | 84.3  15.7 |
| **Ever use modern FP method**  No  Yes | 133  689 | 16.2  83.8 |
| **Duration of modern FP method use**  1-4 years  >=5 years | 530  159 | 76.9  23.1 |
| **Family history of cervical cancer**  No  Yes | 725  97 | 88.2  11.8 |
| **Ever had got pregnant**  No  Yes | 127  695 | 15.5  84.5 |
| **Gravidity**  1-5  >5 | 529  166 | 76.1  23.9 |
| **Ever had gave birth**  No  Yes | 159  663 | 19.3  80.7 |
| **Parity**  1-5  >5 | 558  105 | 84.2  15.8 |

## Knowledge of women on cervical cancer and its screening

More than half of the respondents (59%) were knowledgeable about cervical cancer and its screening (Table 3).

Table 3: Knowledge about cervical cancer and its screening among women (n=822) in Debremarkos town, Northwest Ethiopia, 2018

| **Variable** | **Frequency** | **Percent** |
| --- | --- | --- |
| **Ever heard about cervical cancer**  No  Yes | 130  692 | 15.8  84.2 |
| **Ever heard about cervical cancer screening**  No  Yes | 130  692 | 15.8  84.2 |
| **Cervical cancer screening can be practiced**  No  Yes | 327  495 | 39.8  60.2 |
| **Know Health institutions that gives cervical cancer screening service**  No  Yes | 388  434 | 47.2  52.8 |
| **Do you know symptoms of cervical cancer**  No  Yes | 740  82 | 90.0  10.0 |
| **Bleeding during sexual intercourse may be one of sign of cervical cancer** NoYes  No  Yes | 740  82 | 90.0  10.0 |
| **Cervical cancer is a killer disease**  No  Yes | 281  541 | 34.2  65.8 |
| **Is cervical cancer preventable disease**  No  Yes | 379  443 | 46.1  53.9 |
| **Is cervical cancer curable disease if detected early**  No  Yes | 366  456 | 44.5  55.5 |
| **May have cervical cancer without any sign and symptom**  No  Yes | 480  342 | 58.4  41.6 |

## Attitude towards cervical cancer and its screening

More than half of the respondents (57.4%) had favorable attitude towards cervical cancer and its screening (Table 4).

Table 4: Attitude towards cervical cancer and its screening among women (n=822) in Debremarkos town, Northwest Ethiopia, 2018

| **Variables** | **Level of agreement** | | | | | |
| --- | --- | --- | --- | --- | --- | --- |
|  | **Agree** | | **Disagree** | | **Indifferent** | |
|  | number | percent | number | percent | number | percent |
| Any reproductive age woman is susceptible to develop cervical cancer cance r. | 390 | 47.4 | 225 | 27.4 | 207 | 25.2 |
| Like any women you are susceptible to develop cervical cancer | 372 | 45.3 | 248 | 30.2 | 202 | 24.6 |
| Cervical Cancer is family disease | 111 | 13.5 | 454 | 55.2 | 257 | 31.3 |
| Cervical cancer may be hazardous to your health | 481 | 58.9 | 180 | 22 | 156 | 19.1 |
| Precancerous cervical screening may be beneficial to your wellbeing | 481 | 58.9 | 180 | 22 | 156 | 19.1 |
| Cervical cancer screening is painful | 436 | 53.4 | 153 | 18.6 | 233 | 28.3 |

## Utilization of cervical cancer screening

Forty four (5.4%) of the study population have utilized cervical cancer screening at least once within three years.

**Factors associated with utilization of cervical cancer screening**

Binary logistic regression analysis was employed to identify association between independent and dependent variables using odds Ratio and Adjusted Odds Ratio with 95% Confidence Interval. After controlling the effect of other variables, age of mothers marital status, educational status, age at first sex, history of MSP, history of STD, duration of FP use, family history of CC, gravidity, knowledge and attitude about CC and its screening continued to be significantly associated with utilization of cervical cancer screening (P-values<0.2).

Multivariable logistic regression analysis was used to control the effect of confounders. After controlling the effect of other variables, Women’s age [AOR: 3.126 (95% CI: 1.246, 7.845)], marital status [AOR: 3.41 (95% CI: 1.299, 8.972)], education level (secondary education level [AOR: 4.578(95% CI: 1.19, 17.65)] and College and above education level [AOR:7.27,95%CI: 2.07,25.513)]), age at first sexual intern course [AOR:3.021(95%CI:1.84, 4.97)], history of MSP[AOR:2.51 (95% CI:1.040, 6.06)], history of sexually transmitted disease [AOR:4.04(95% CI:1.68, 9.72)], knowledge about cervical cancer and screening [AOR:4.02,95% CI:2.07, 7.77)] and attitude about cervical cancer and screening [AOR:3.23,95%CI;2.52, 4.12)] were significant factors for utilization of cervical cancer screening (Table 5 ).

# Discussion

Forty four (5.4%) of the study population have been utilized cervical cancer screenings a minimum of once within the last three years with [95% CI: 3.8, 7.1] as compared to different studies.

The study finding of this research was in-line with similar report in Arbaminch town, Southern Ethiopia(5.9%)(16).

The finding of this research was lower than the studies done: at Addis Ababa, Ethiopia(10.8%) (17), at Mekelle town, Northern Ethiopia(19.8%) (15), at Hadiya Zone, South Ethiopia (9.9%)(18), in Ethiopia (17%) (19), Kenya(16%) (20), Nigeria (11%) (21).

Table 5*:* Bivariable and Multivariable analysis of factors associated with utilization of cervical cancer screening among women in Debremarkos town, Northwest Ethiopia, 2018

| **Variable** | **Utilized** | | | **Crude OR[95%CI]** | **AOR[95%CI]** |
| --- | --- | --- | --- | --- | --- |
|  | **Yes** | **No** | |  |  |
| **Age of mothers**  40-49  30-39 | 31  13 | | 199  579 | **6.938(3.56,13.52)**  **1** | **3.126(1.246,7.85)**  **1** |
| **Marital status**  Others**  Married | 15  29 | | 169  609 | **1.864(1.09,3.557)**  **1** | **3.41 (1.299,8.97)**  **1** |
| **Religion**  Muslim  Christian | 1  43 | | 32  746 | 0.542(0.072,4.062)  1 |  |
| **Educational status**  Primary education  Secondary education  College and above  No formal education | 7  10  20  7 | | 173  202  179  224 | 1.295 (.446,3.761)  **1.584(1.059,4.240)**  **3.575(1.479,8.645)**  **1** | **4.578(1.19,17.65)**  **7.27(2.07,25.51)***  **1** |
| **Ethnicity**  Others** *  Amhara | 1  43 | | 12  766 | 1.484(.189,11.682)  1 |  |
| **Occupation**  Self-employee  Private employee  Government employee  House wife | 14  16  8  6 | | 295  176  164  143 | 1.131(1.426,3.005)  2.167(.826,5.681)  1.163(.394,3.430)  1 |  |
| **House hold income**  900–1600  1601–2699  >=2700  <900 | 10  18  11  5 | | 191  275  151  161 | 1.686(.565,5.033)  2.108(.768,5.785)  2.346(.796,6.909)  1 |  |
| **Age at first sex**  <=16  >16 | 24  20 | | 177  601 | **4.075(2.483,6.717)**  **1** | **3.021(1.84,4.971)**  **1** |
| **Multiple sexual partner**  Yes  No | 30  14 | | 331  447 | **2.894(1.511,5.544)**  **1** | **2.511 (1.04,6.06)**  **1** |
| **History of smoking**  Yes  No | 2  42 | | 15  763 | 2.422(.536,10.94)  1 |  |
| **History of STD**  Yes  No | 18  26 | | 111  667 | **4.160(2.208,7.840)**  **1** | **4.037(1.68,9.72)***  **1** |
| **Variable** | **utilized** | | | **Crude OR[95%CI]** | **AOR[95%CI]** |
|  | **Yes** | **No** | |  |  |
| **Ever use modern FP method**  Yes  No | 34  10 | | 655  123 | .638(.307,1.326)  1 |  |
| **Duration of modern FP method usage**  >=5 years  1-4 years | 18  19 | | 141  511 | 3.433(1.746,6.68)  1 | 1.771(.711,4.41)  1 |
| **Family history of cervical cancer**  Yes  No | 8  36 | | 89  689 | 1.720(1.041,4.218)  1 | 1.897(.701,5.134)  1 |
| **Ever had got pregnant**  Yes  No | 39  5 | | 656  122 | 1.451 (.517,3.101)  1 |  |
| **Gravidity**  >5  1-5 | 19  19 | | 147  510 | 3.469(1.790, 6.72)  1 | 1.421(.58, 3.47)  1 |
| **Ever had gave birth**  Yes  No | 35  9 | | 628  150 | .929(.437, 1.974)  1 |  |
| **Parity**  >5  1-5 | 7  30 | | 98  528 | 1.257(.540,2.958)  1 |  |
| **Knowledge**  knowledgeable  not knowledgeable | 31  13 | | 454  324 | **1.702(1.01,3.29)**  **1** | **4.02(2.07, 7.77)**  **1** |
| **Attitude**  favorable attitude  unfavorable attitude | 33  11 | | 439  339 | **2.317(1.81, 2.96)**  **1** | **3.225(2.52, 4.12)**  **1** |

*^p value less than 0.01,^ ** ^Single, divorced and widowed,^ ***^Oromo and Guragie^

The possible explanation for this might be: the difference in the age of study population as evidenced by studies done at Mekelle town with the age greater than 21 years, Addis Ababa from 21 to 64 years , Hadiya Zone from 18 to 49 years , Kenya with age greater than 18 years ; the difference in study area as evidenced by studies done at Addis Ababa ,Ethiopia, Nigeria which were institution based; the difference health status of the population as evidenced by the study done at Addis Ababa on human immune deficiency virus (HIV) patients; the difference with sample size as evidenced by studies done at Mekelle town; the difference with the level of knowledge and attitude of study population as evidenced by studies done in Ethiopia on health care providers (HCPs), in Nigeria among teaching hospital staffs.

Again this study finding was higher than studies done in Ethiopia (2.9%) (22) and Southern Ghana (0.8%) (23). The possible explanation might be: the difference in study area and study population as evidenced by study done in Ethiopia on both urban and rural population that was different from our study population and study area which was urban; difference in the age of the study population as evidenced by study done in Ghana which included 10 to 74 years which showed the probability of being utilized was lower since the respondents were too young and too old which were not eligible for utilization of screening service.

As shown from this study, mother’s age was one of the significant factors of utilization of cervical cancer screening. Women in their age 40-49 years were 3.126 times more likely utilized cervical cancer screening as compared to women in their age of 30-39 years [AOR:3.126(95% CI:1.246, 7.845)]. This finding was supported with the studies done in Northern Ethiopia, Addis Ababa, Southern Ethiopia, Ethiopia and Malawi (15, 16, 22, 24, 25). The possible explanation for this might be as women’s age increases the probability being exposed for cervical cancer risk factors will be increased which leads them utilized cervical cancer screening service. The other explanation also might be as the women’s age increases, the knowledge and attitude about the disease and its screening might be increased.

Marital status was also one of the significant predictor for utilization of cervical cancer screening. This study showed that, women who were Single/divorced/widowed 3.414 times more likely utilized cervical cancer screening as compared with married women [AOR:3.414,(95%CI:1.299,8.972)]. This result was supported with the study done in Thailand (26). The possible explanation for this might be single women, divorced women or widowed women may have MSP which are the risk for STD and its symptom that increases the chance of utilizing cervical cancer screening. On the other hand, women who were married may not think as they are susceptible for cervical cancer.

Educational status was also the main significant factor for utilization cervical cancer screening. Women who took college & above education 7.268 times more likely utilized as compared with women who did not take formal education [AOR: 7.268(95% CI:2.071,25.513)].Similarly Women who attended secondary education 4.578 times more likely utilized as compared with women who did not attended formal education [AOR: 4.578 (95% CI: 1.187,17.649)].This study was supported with the study done Ethiopia and Kenya (20, 22). This might be explained with, as level of education increases, the women will have the chance to have knowledge about cervical cancer and its screening that makes the women utilizing the screening service. This shows women who have high level of education may have the probability to know risk factors and preventives of cervical cancer.

Age at first sexual inter course was the significant predictor for utilization of cervical cancer screening. Women who had started sex with their age of sixteen years and below were 3.021 times more likely utilized cervical cancer screening as compared to those women who had started sex after their age of sixteen years [AOR: 3.021 (95%CI; 1.84, 4.97)]. The possible explanation for this might be women, who started sex with early age; the probability to have life time sexual partner might be increased which increases the chance of being infected with sexual transmitted infection (STI) and STD that increases sign and symptoms of these diseases which makes them to utilize cervical cancer screening; the other explanation might be also if women start sex with early age, since cervical cell is immature, the probability of getting of cervical cancer cells change may be occur at early due to sperm contact and the sign and symptom might be occurred which leads for utilization of cervical cancer screening; no literature found to compare with this study because, it is sensitive issue information biases might be occurred which was tried to avoid this biases in our study with care full interviewing technique.

This study finding showed that mother who had history of MSPs was other important factor for utilization of cervical cancer screening. Women who have had history of MSPs were 2.51 times more likely utilized cervical cancer screening as compared to those who did not MSP [AOR: 2.51(95% CI: 1.040, 6.062)]. This study was supported with the result of a study done in Addis Ababa, at Mekelle, Malawi and Thailand (15, 24-26). The possible explanation might be women who had history MSPs might suspect themselves as they are risked for STIs and may have a chance utilize cervical cancer screening. The other explanation might be women, who had MSP history, would have the chance to get STI infections with its sign and symptoms that makes them to consult HCPs which increases the utilization of cervical cancer screening service.

The current study result showed that mother who had history of STD was other important factor of utilization of cervical cancer screening. Women who have had history of STD were 4.037 times more likely utilized cervical cancer screening as compared to those who did not have STD history [AOR: 4.037, (95%Cl: 1.68, 9.72)].This result was supported with the finding from Addis Ababa and Northern Ethiopia (15, 24).The above association might be explained by women who have STD and history of STD, will have increased chance of visiting health institutions for treatment and for other medical help that increases the chance of utilization of cervical cancer screening service.

Women’s knowledge about cervical cancer and its screening was another significant factor for utilization of cervical cancer screening service. Women who were knowledgeable about cervical cancer and its screening were 4.02 times more likely utilized cervical cancer screening service as compared to those who were not knowledgeable[AOR:4.02 (95% CI:2.07, 7.77)]. This finding was supported with the result of studies done in Northern Ethiopia, Malawi, Tanzania and Thailand (15, 25-27).The above reports might be explained with increased level of women’s knowledge about the risk factors of cervical cancer and its screening increases the chance of utilizing cervical cancer screening. If mothers know about severity of cervical cancer and benefits of screening and risk factors of the disease, they will have high chance to utilize cervical cancer screening.

Similarly, women’s attitude about cervical cancer and its screening was also significant factor for utilization of cervical cancer screening service. Women who had favorable attitude about cervical cancer and its screening were 3.225 times more likely utilized cervical cancer screening service as compared to those who had unfavorable attitude [AOR:3.225 (95%CI:2.52, 4.12)].This study was supported by a study done in Northern Ethiopia, Southern Ethiopia ,Southern Ghana and Thailand (15, 16, 23, 26).The above reports might be explained with women who have favorable attitude towards cervical cancer and its screening will have self-initiative to know about cervical cancer risk factors and benefits of its screening that makes them to utilize cervical cancer screening.

# Conclusion

This study showed the magnitude of utilization of cervical cancer screening was low. Age of the women, marital status, and educational status, age at first sexual intercourse, history of multiple sexual partners and sexually transmitted disease, knowledge and attitude were statistically significant factors of utilization of cervical cancer screening.

# Acknowledgements

We would like to thank University of Gondar and Debremarkos health office for their permission to this research and we gratefully acknowledge all study individuals for their participation in the study.

# Authors’ contributions

BY: proposed the initial idea for the study; BY, KT, MM: contributed to the study design; BY: collected all the data; all authors analyzed and interpreted the data; BY: drafted the manuscript; BY, KT and MM: contributed to the writing of the manuscript. BY, KT and MM: prepared the manuscript for publication; all authors read and approved the final manuscript.

# References

1. WHO U, PATH. cryosurgical equipment for the treatment of precancerous cervical lesions and prevention of cervical cancer 2012.
2. WHO/NMH/NMA. UN Joint Global Programme on Cervical Cancer Prevention and Control. The United Nations Global Cervical Cancer Programme 2016.
3. Bruni L B-RL, AlberoG, SerranoB, Mena M, GómezD, Muñoz J, Bosch FX, de Sanjosé S.ICO/IARC Information Centre on HPV and Cancer (HPV Information Centre). Human Papillomavirus and Related Diseases in Ethiopia. 2017.
4. Organization WH. WHO guidelines for screening and treatment of precancerous lesions for cervical cancer prevention. 2013.
5. WHO. WHO guidance note:comprehensive cervical cancer prevention and control: a healthier future for girls and women. 2013.
6. Leslea Peirson* DF-L, Donna Ciliska and Rachel Warren. Screening for cervical cancer: a systematic review and meta analysis. Systematic Review. 2013;2(35).
7. Control CfD, Prevention. Cervical cancer screening among women aged 18-30 years-United States, 2000-2010. MMWR Morbidity and mortality weekly report. 2013;61(51-52):1038.
8. EVA JOHANNA KANTELHARDT a, b ULRIKEMOELLE,a MATTHIAS BEGOIHN,a ADAMU ADDISSIE,c PIETRO TROCCHI,b BEKURETSION YONAS,d, PETROS HEZKIEL eAS, b,g CHRISTOPH THOMSSEN,a DIRK VORDERMARK,c TUFA GEMECHU,d YIRGU GEBREHIWOT,e, TIGENEHWONDEMAGEGNEHU fAA, f ASSEFAMATHEWOSf. Cervical Cancer in Ethiopia: Survival of 1,059 Patients Who Received Oncologic Therapy. 2014;9:1727–734.
9. WHO. Prevention of cervical cancer through screening using visual inspection with acetic acid (VIA) and treatment with cryotherapy 2012.
10. foundationforwomenscancer.org. understanding cervical cancer 2013.
11. FMOH. National cancer control plan of Ethiopia 2015.
12. ASCCP. Cervical Cancer Screening Recommendations 2012.
13. Catarino R, Petignat P, Dongui G, Vassilakos P. Cervical cancer screening in developing countries at a crossroad: Emerging technologies and policy choices. World Journal of Clinical Oncology. 2015;6(6):281-90.
14. De Sanjosé S, Serrano B, Castellsagué X, Brotons M, Muñoz J, Bruni L, et al. Human papillomavirus (HPV) and related cancers in the Global Alliance for Vaccines and Immunization (GAVI) countries. A WHO/ICO HPV Information Centre Report. Vaccine. 2012;30(Suppl 4):D1-83.
15. Bayu H, Berhe Y, Mulat A, Alemu A. Cervical cancer screening service uptake and associated factors among age eligible women in Mekelle Zone, Northern Ethiopia, 2015: a community based study using health belief model. PLoS One. 2016;11(3):e0149908.
16. Gebru Z, Gerbaba M, Dirar A. Utilization of Cervical Carcinoma Screening Service and Associated Factors among Currently Married Women in Arba Minch Town, Southern Ethiopia. J Women’s Health Care. 2016;5(297):2167-0420.1000297.
17. Shiferaw S, Addissie A, Gizaw M, Hirpa S, Ayele W, Getachewe S, et al. Knowledge about cervical cancer and barriers toward cervical cancer screening among HIV-positive women attending public health centers in Addis Ababa city, Ethiopia. Cancer Medicine. 2016:n/a-n/a.
18. Aweke YH, Ayanto SY, Ersado TL. Knowledge, attitude and practice for cervical cancer prevention and control among women of childbearing age in Hossana Town, Hadiya zone, Southern Ethiopia: Community-based cross-sectional study. PLOS ONE. 2017;12(7):e0181415.
19. Kress CM, Sharling L, Owen-Smith AA, Desalegn D, Blumberg HM, Goedken J. Knowledge, attitudes, and practices regarding cervical cancer and screening among Ethiopian health care workers. International Journal of Women's Health. 2015;7:765-72.
20. Morema EN, Atieli HE, Onyango RO, Omondi JH, Ouma C. Determinants of cervical screening services uptake among 18–49 year old women seeking services at the Jaramogi Oginga Odinga Teaching and Referral Hospital, Kisumu, Kenya. BMC health services research. 2014;14(1):335.
21. Eze GU, Obiebi IP, Umuago IJ. Perspectives of cervical cancer and screening practices among staff of a teaching hospital in South-South Nigeria. Journal of Cancer Research and Practice. 2018.
22. Terefe Gelibo1, * LR, TG, Bekele3 aA. Coverage and Factors Associated with Cervical Cancer Screening: Results from a Population-Based WHO Steps Study in Ethiopia. Journal of Oncology Research and Treatments. 2017;2(115).
23. Ebu NI, Mupepi SC, Siakwa MP, Sampselle CM. Knowledge, practice, and barriers toward cervical cancer screening in Elmina, Southern Ghana. International Journal of Women's Health. 2015;7:31-9.
24. Teame H, Addissie A, Ayele W, Hirpa S, Gebremariam A, Gebreheat G, et al. Factors associated with cervical precancerous lesions among women screened for cervical cancer in Addis Ababa, Ethiopia: A case control study. PLOS ONE. 2018;13(1):e0191506.
25. Chosamata MS, Hong SA, Tiraphat S. Determinants of cervical cancer screening utilization among women aged 30-45 years in Blantyre district, Malawi. 2015.
26. Visanuyothin S, Chompikul J, Mongkolchati A. Determinants of cervical cancer screening adherence in urban areas of Nakhon Ratchasima Province, Thailand. Journal of infection and public health. 2015;8(6):543-52.
27. Lyimo FS, Beran TN. Demographic, knowledge, attitudinal, and accessibility factors associated with uptake of cervical cancer screening among women in a rural district of Tanzania: three public policy implications. BMC public health. 2012;12(1):22.
