# Supplementary material for: Utilization of cervical cancer screening and associated factors among women in Debremarkos town, Amhara region, Northwest Ethiopia: Community based cross-sectional study
Source: PLoS One. 2020 Apr 7;15(4):e0231307. doi: 10.1371/journal.pone.0231307 (PMC7138328; doi:10.1371/journal.pone.0231307)
Supplement: Attachment — Submitted filename: Response to Reviewers.docx [file pone.0231307.s003.docx]

**Response to Reviewers**

Dear reviewer, thank you for your constructive comments on our manuscript.

- For your first question "Why Adjusted Odds Ratio with p-value pf 0.2?” we have edited and corrected in the 2^nd^ submission after the first comment. And some statistics books said to use "p-value" cut off point 0.2 and others 0.25. But we have used "p-value" 0.2 to include more variables.
- “How inline is inline?” since the confidence interval of may study result[95% CI: 3.8, 7.1] includes other study findings with similar research questions we have used the term **in line with.**
- The age classification is made after we reviewed related literatures. So, the category is based on different literatures.
- In **Marital status,** we have merged “Single, divorced and widowed”, and replaced with the title "others" to avoid a small value in a cell
- **Similarly, in Religion** and **Ethnicity, we merged corresponding variables to minimize small value occurrence in a single cell.**
- Categorizing **“Household income" is** based on similar literatures.
