# Supplementary material for: Utilization of cervical cancer screening and associated factors among women in Debremarkos town, Amhara region, Northwest Ethiopia: Community based cross-sectional study
Source: PLoS One. 2020 Apr 7;15(4):e0231307. doi: 10.1371/journal.pone.0231307 (PMC7138328; doi:10.1371/journal.pone.0231307)
Supplement: Attachment — Submitted filename: Response to reviewers.docx [file pone.0231307.s005.docx]

**Response to reviewers**

**Thank you for your constructive comments‼ Here are some responses for your questions.**

1. For your question “**how could you analyze all the factors related with service use**?” We have analyzed by using logistic regression model (binary and multi variable), i.e. Bivariate logistic regression was employed to identify an association between independent and dependent variables. Variables having a P-value of less than 0.2 in the bivariate logistic regression analysis were fitted into the multivariable logistic regression model. The 95% confidence interval of odds ratio was computed and variable having P-value less than 0.05 in the multivariable logistic regression analysis was considered as statistically significant.
2. For your comment “ **what are the rigorous efforts made to overcome the problem, and what are the gap still existed, what are the contribution of your study going to fill the gap**” efforts have been listed on the manuscript(Ethiopia adopted the WHO guideline and advised women to start cervical cancer screening at age of 30-49 years at least one to three years interval with the approach of seeing and treat through Visual Inspection under Acetic acid (VIA) as screening strategy and cryo-therapy as a treatment method(1-4).”

And gaps have been identified (a high incidence of cervical cancer is still a big problem and a major cause of morbidity and mortality of women in LMICs especially in SSA (5, 6) and the lowest cervical cancer screening rate found in Ethiopia which accounts below 1% (7)).

Again the main contributions to fill gaps have been mentioned (Therefore, the main aim of this research was to identify factors affecting cervical screening utilization and recommend ways to increase screening utilization by the community).

3. For your Q “**why this area has been selected for this particular study?**” When we were health care providers in Debremarkos hospital at different time, there were many cases presented with advanced stage of cervical cancer and more than half of the case was from Debremarkos town which makes us to do this research. The other reason is there was no research with the same study design and study population on CC screening and its associated factors.
4. For Q “**Why 30-49? As WHO recommended from 30-50?**” Ethiopian ministry of health recommended screening from 30-49 years, since the gap between pre cervical lesion and cervical cancer takes 10 to 20 years, screening mothers aged ≥50 years is not that much important?
5. For Q “**what is your sampling interval? The number of kth value? How did you calculate it?** Tell us please that number?” since we have used systematic sampling technique we have calculated the sampling interval (k^th^ value) for each kebele with formula of N/n=k (sampling interval). The kth value for each kebele was 14(k2=N/n =4681/328=14.2~14; k3=N/n=4529/318=14.2~14; k5=N/n=2501/176=14.2~14)
6. **For Q “What are the potential biases that you tried to reduce during your study period?**” we have tried to reduce selection biases by using improved sampling technique. Three out of the seven kebeles were selected by the lottery method. Again the list of households found and coded in each kebele. The size of households consisting of eligible population to be selected from each kebele was determined proportionally based on the size of the study units and the k^th^ value was computed for each selected kebele. If there was more than one woman in the household during sampling, the lottery method has been used to select only one. In the case of absenteeism, after three repeated visits the next eligible woman was included in the study.

We have tried to reduce information biases by using well trained data collectors, avoiding leading questions, educating the purpose and the importance of the research, reducing screened time (included to the study we utilized screening within 3 years)

**For comment** “Please use the references for this number that you used for bivariate analysis”. We have used 0.2 by referring published articles (8, 9)

7. For your Q” **What best practice could you share us that helped you to attain 100% response rate?”** we have provided education about the importance this research and the main purpose of the research. All study participants were happy to give the response since they were clear with the purpose and its importance.
8. For Q “**What is special significance of these two variables (Ethnicity and religion)?”** Since different ethnic group with different religion followers live in Debremarkos town and participants from some ethnic origin may perceive cervical cancer screening bad culture and their response may affect the study result. Again cervical cancer screening may be forbidden in some religions and participants’ response from these religions may affect the study result.

9. For Q **“ What mechanism did you use to investigate the Social desirability bias”** we have informed well the study participants about the confidentiality and the questionnaire do not concern on the private information and taken as anonymously. Again full purpose of the research has been informed for them.

1.	FMOH. National cancer control plan of Ethiopia. 2015.

```
2.	Organization WH. WHO guidelines for screening and treatment of precancerous lesions for cervical cancer prevention. 2013.
3.	WHO. WHO guidance note:comprehensive cervical cancer prevention and control: a healthier future for girls and women. 2013.
4.	WHO U, PATH. cryosurgical equipment for the treatment of precancerous cervical lesions and prevention of cervical cancer. 2012.
5.	ASCCP. Cervical Cancer Screening Recommendations. 2012.
6.	Catarino R, Petignat P, Dongui G, Vassilakos P. Cervical cancer screening in developing countries at a crossroad: Emerging technologies and policy choices. World Journal of Clinical Oncology. 2015;6(6):281-90.
7.	De Sanjosé S, Serrano B, Castellsagué X, Brotons M, Muñoz J, Bruni L, et al. Human papillomavirus (HPV) and related cancers in the Global Alliance for Vaccines and Immunization (GAVI) countries. A WHO/ICO HPV Information Centre Report. Vaccine. 2012;30(Suppl 4):D1-83.
8.	Teame H, Addissie A, Ayele W, Hirpa S, Gebremariam A, Gebreheat G, et al. Factors associated with cervical precancerous lesions among women screened for cervical cancer in Addis Ababa, Ethiopia: A case control study. PLOS ONE. 2018;13(1):e0191506.
9.	Getinet M, Gelaw B, Sisay A, Mahmoud EA, Assefa A. Prevalence and predictors of Pap smear cervical epithelial cell abnormality among HIV-positive and negative women attending gynecological examination in cervical cancer screening center at Debre Markos referral hospital, East Gojjam, Northwest Ethiopia. BMC Clinical Pathology. 2015;15:16.
```
